# Supplementary material for: Benchmarking Differential Abundance Tests for 16S microbiome sequencing data using simulated data based on experimental templates
Source: PLoS One. 2025 May 19;20(5):e0321452. doi: 10.1371/journal.pone.0321452 (PMC12088514; doi:10.1371/journal.pone.0321452)
Supplement: S1 Table — These are used to calculate the final 46 scalar value characteristics (S2 Table). (PDF) [file pone.0321452.s001.pdf]

S1 Table: Higher dimension data characteristics. These are used to calculate the final 46 scalar value characteristics (S2 Table)

Name of data characteristic	Element names in the collective data characteristics list data.prop	Calculation in R	Dimension
dat.cpm<br>Counts per million normalized and log transformed data		edgeR::cpm(dat, log=TRUE, prior.count = 1)	mxn
Feature sparsity	data.prop$P0_feature	apply(dat==0,1,sum)/ncol(dat)	m
Sample sparsity	data.prop$P0_sample	apply(dat==0,2,sum)/nrow(dat)	n
Feature mean abundance	data.prop$mean_log2cpm	apply(dat.cpm, 1,mean,na.rm=T)	m
Feature median abundance	data.prop$median_log2cpm	apply(dat.cpm,1, median,na.rm=T)	m
Feature variance	data.prop$var_log2cpm	apply(dat.cpm, 1, var)	m
Library size	data.prop$lib_size	colSums(dat)	n
Sample means	data.prop$sample_means	apply(dat,2,mean)	n
Sample correlation	data.prop$corr_sample	cor(dat, dat, method="spearman",use="na.or.complete")	nxn

Feature correlation	data.prop$corr_feature	cor(t(dat), t(dat), method="spearman",use="na.or.complete")	mxm

Data characteristics in the form of vectors or matrices that are calculated from (mxn)-count data matrix *dat*, with features as rows and samples as columns.
