# Supplementary material for: Benchmarking Differential Abundance Tests for 16S microbiome sequencing data using simulated data based on experimental templates
Source: PLoS One. 2025 May 19;20(5):e0321452. doi: 10.1371/journal.pone.0321452 (PMC12088514; doi:10.1371/journal.pone.0321452)
Supplement: S2 Table — (PDF) [file pone.0321452.s002.pdf]

S2 Table: Final integer values data characteristic

Name of data characteristic	Calculation in R
Number of features	nrow(dat)
Number of samples	ncol(dat)
Sparsity of dataset	sum(dat==0)/length(dat)
Median of dataset	median(dat,na.rm=TRUE)
95th Quantile	quantile(dat,probs=.95)
99th Quantile	quantile(dat,probs=.99)
Mean library size	mean(colSums(dat),na.rm = T)
Median library size	median(colSums(dat),na.rm = T)
Standard deviation library size	sd(colSums(dat),na.rm = T)

Coefficient of variation of library size	sd(colSums(dat),na.rm = T)/mean(colSums(dat),na.rm = T)*100
Maximum library size	max(colSums(dat),na.rm = T)
Minimum library size	min(colSums(dat),na.rm = T)
Read depth range between samples	diff(range(colSums(dat),na.rm = T))
Mean sample richness	mean(colSums(dat>0), na.rm=T)
Spearman correlation library size with P0*(sample)	cor(data.prop$P0_sample, data.prop$lib_size, method="spearman")
Bimodality of feature correlations	bimodalIndex(matrix(data.prop$corr_feature,nrow=1))$BI
Bimodality of sample correlations	bimodalIndex(matrix(data.prop$corr_sample,nrow=1))$BI
Mean of all feature means	mean(data.prop$mean_log2cpm,na.rm=T)
SD of all feature means	sd(data.prop$mean_log2cpm,na.rm=T)
Median of all feature means	median(data.prop$median_log2cpm,na.rm=T)
SD of all feature medians	sd(data.prop$median_log2cpm,na.rm=T)

Mean of all feature variances	mean(data.prop$var_log2cpm,na.rm=T)
SD of all feature variances	sd(data.prop$var_log2cpm,na.rm=T)
Mean of all sample means	mean(data.prop$sample_means,na.rm=T)
SD of all sample means	sd(data.prop$sample_means,na.rm=T)
Mean of sample correlation matrix	mean(data.prop$corr_sample,na.rm=T)
SD of sample correlation matrix	sd(data.prop$corr_sample,na.rm=T)
Mean of feature correlation matrix	mean(data.prop$corr_feature,na.rm=T)
SD of feature correlation matrix	sd(data.prop$corr_feature,na.rm=T)
Mean-Variance relation: Linear component	res =lm(y~x+I(x^2),data= data.frame(y=data.prop$var_log2cpm,x=data.prop$mean_log2cpm)) res$coefficients[2]

Mean-Variance relation: Quadratic component	res=lm(y~x+I(x^2),data=data.frame(y=data.prop$var_log2cpm,x=data.prop$mean_log2cpm))<br><br>res$coefficients[3]
Slope feature sparsity vs. feature mean	res=lm(y~slope,data=data.frame(slope=data.prop$P0_feature-1,y=data.prop$mean_log2cpm))<br><br>res$coefficients[2]
Clustering of features	coef.hclust(hcluster(dat.tmp))
Clustering of samples	coef.hclust(hcluster(t(dat.tmp)))
Sample sparsity	apply(dat==0,2,sum)/nrow(dat)
Library sizes	colSums(dat)
Mean read depths	apply(dat,2,mean)
Feature sparsity	apply(dat==0,1,sum)/ncol(dat)
Feature mean intensity	apply(dat.cpm, 1,mean)
Feature median intensity	apply(dat.cpm,1, median)
Feature variances	apply(dat.cpm, 1, var)
Sample correlations	cor(dat, dat, method="spearman")
Feature correlations	calc_feature_corr(dat)
Mean inverse Simpson diversity	mean(vegan::diversity(dat, index = "invsimpson"),na.rm=T)

Mean Pilou evenness	shannon_div <- vegan::diversity(count.data, index = "shannon")<br><br>richness <- apply(count.data, 1, function(x) sum(x > 0,na.rm = T))<br><br>pilou <- shannon_div / log(richness)<br><br>mean(pilou (dat),na.rm=T)
Mean Bray-Curtis dissimilarity	mean(vegan::vegdist(dat, method = "bray"),na.rm=T)

*P0: Percent of zeros
