# Supplementary material for: Benchmarking Differential Abundance Tests for 16S microbiome sequencing data using simulated data based on experimental templates
Source: PLoS One. 2025 May 19;20(5):e0321452. doi: 10.1371/journal.pone.0321452 (PMC12088514; doi:10.1371/journal.pone.0321452)
Supplement: S3 Table — (PDF) [file pone.0321452.s003.pdf]

S3 Table: Summary information on 38 experimental data templates, which serve as templates for calibrating the simulation tool.

Dataset	Name in Nearing et al.	Number of features	Number of samples	Meta data	Number of conditions	Sparsity
ArcticFireSoils	ArcticFireSoils	8148	147	condition	2	0,866
ArcticFreshwaters	ArcticFreshwaters	33045	2296	condition	2	0,991
ArcticTransects	ArcticTransects	20959	197	condition	2	0,926
art_scher	art_scher	1171	69	condition	2	0,845
asd_son	asd_son	2726	102	condition	2	0,866
BISCUIT	BISCUIT	567	38	condition	2	0,826
Blueberry	Blueberry	6903	63	condition	2	0,83
cdi_schubert	cdi_schubert	2164	237	condition	2	0,905
cdi_vincent	cdi_vincent	575	40	condition	2	0,791
Chemerin	Chemerin	515	115	condition	2	0,758
crc_baxter	crc_baxter	16268	263	condition	2	0,953
crc_zeller	crc_zeller	35029	116	condition	2	0,887
edd_singh	edd_singh	954	203	condition	2	0,899
Exercise	Exercise	1175	81	condition	2	0,761
glass_plastic_oberbeckmann	glass_plastic_oberbeckmann	730	30	condition	2	0,768
GWMC_ASIA_NA	GWMC_ASIA_NA	34144	864	condition	2	0,921
GWMC_HOT_COLD	GWMC_HOT_COLD	36799	1021	condition	2	0,928
hiv_dinh	hiv_dinh	622	27	condition	2	0,739
hiv_lozupone	hiv_lozupone	612	27	condition	2	0,703
hiv_noguerajulian	hiv_noguerajulian	11749	170	condition	2	0,904
ibd_gevers	ibd_gevers	6913	154	condition	2	0,886
ibd_papa	Human - IBD	1331	24	condition	2	0,795
Ji_WTP_DS	Ji_WTP_DS	327	59	condition	2	0,769
MALL	MALL	407	36	condition	2	0,834
ob_goodrich	ob_goodrich	53280	613	condition	2	0,957
ob_ross	ob_ross	927	52	condition	2	0,776
ob_turnbaugh	ob_turnbaugh	4538	78	condition	2	0,879
ob_zhu	ob_zhu	2286	41	condition	2	0,799
Office	Office	8875	1174	condition	2	0,987
par_scheperjans	par_scheperjans	2305	108	condition	2	0,897
sed_plastic_hoellein	sed_plastic_hoellein	3870	31	condition	2	0,749
sed_plastic_rosato	sed_plastic_rosato	1064	39	condition	2	0,635
seston_plastic_mccormick	seston_plastic_mccormick	3706	65	condition	2	0,834
sw_plastic_frere	sw_plastic_frere	1896	59	condition	2	0,759

sw_sed_detender	sw_sed_detender	3276	78	condition	2	0,86
t1d_alkanani	t1d_alkanani	59736	75	condition	2	0,925
t1d_mejialeon	t1d_mejialeon	388	28	condition	2	0,653
wood_plastic_kesy	wood_plastic_kesy	1685	72	condition	2	0,781

Size, sparsity and meta information summary for the experimental templates.
